# HMGA1 overexpression in adipose tissue impairs adipogenesis and prevents diet-induced obesity and insulin resistance

**DOI:** 10.1038/srep14487

**Published:** 2015-09-28

**Authors:** Altamira Arce-Cerezo, Miquel García, Aida Rodríguez-Nuevo, Mireia Crosa-Bonell, Natalia Enguix, Albert Peró, Sergio Muñoz, Carles Roca, David Ramos, Sylvie Franckhauser, Ivet Elias, Tura Ferre, Anna Pujol, Jesús Ruberte, Josep A. Villena, Fàtima Bosch, Efrén Riu

**Affiliations:** 1 Center of Animal Biotechnology and Gene Therapy, Universitat Autònoma de Barcelona, Bellaterra, Spain; 2 Department of Biochemistry and Molecular Biology, Universitat Autònoma de Barcelona, Bellaterra, Spain; 3 Centro de Investigación Biomédica en Red de Diabetes y Enfermedades Metabólicas Asociadas (CIBERDEM), Barcelona, Spain; 4 Laboratory of Metabolism and Obesity, Vall d’Hebron-Institut de Recerca, Universitat Autònoma de Barcelona, Barcelona, Spain; 5 Department of Anatomy and Animal Health, School of Veterinary Medicine, Universitat Autònoma de Barcelona, Bellaterra, Spain

## Abstract

High-Mobility-Group-A1 (HMGA1) proteins are non-histone proteins that regulate chromatin structure and gene expression during embryogenesis, tumourigenesis and immune responses. *In vitro* studies suggest that HMGA1 proteins may be required to regulate adipogenesis. To examine the role of HMGA1 *in vivo*, we generated transgenic mice overexpressing HMGA1 in adipose tissues. HMGA1 transgenic mice showed a marked reduction in white and brown adipose tissue mass that was associated with downregulation of genes involved in adipogenesis and concomitant upregulation of preadipocyte markers. Reduced adipogenesis and decreased fat mass were not associated with altered glucose homeostasis since HMGA1 transgenic mice fed a regular-chow diet exhibited normal glucose tolerance and insulin sensitivity. However, when fed a high-fat diet, overexpression of HMGA1 resulted in decreased body-weight gain, reduced fat mass, but improved insulin sensitivity and glucose tolerance. Although HMGA1 transgenic mice exhibited impaired glucose uptake in adipose tissue due to impaired adipogenesis, the increased glucose uptake observed in skeletal muscle may account for the improved glucose homeostasis. Our results indicate that HMGA1 plays an important function in the regulation of white and brown adipogenesis *in vivo* and suggests that impaired adipocyte differentiation and decreased fat mass is not always associated with impaired whole-body glucose homeostasis.

Metabolic and endocrine dysfunction of the adipose compartment is central to the pathologies associated with metabolic diseases such as obesity and lipodystrophy, both in animal models and humans^1^,^2^,^3^,^4^. Obesity and lipodystrophy show similar metabolic derangements and both are major risk factors for serious chronic diseases, including insulin resistance, type 2 diabetes, hypertension and cardiovascular disease^5^. Whereas lipodystrophy is a rare disease with a low prevalence, the number of obese individuals has been growing steadily in the occidental world and developing countries and currently represents a considerable economic burden for many healthcare systems^6^,^7^. Since no effective treatments are available^8^ there is urgent need to develop novel and safe anti-obesity therapies.

Obesity is defined as an excess accumulation of white adipose tissue (WAT), resulting from both an increase in adipocyte cell size (hypertrophy) and in the proliferation and differentiation of new adipocytes (hyperplasia), providing more adipocytes for hypertrophic expansion^2^,^9^. Adipocytes differentiate from undifferentiated preadipocytes in a process termed adipogenesis^2^,^10^. Although it is clear that the late stages of adipocyte maturation are governed by the nuclear receptor peroxisome proliferator-activated receptor gamma (*Pparγ*) and CCAAT/enhancer binding protein *alpha* (*C/ebp*α)^2^,^10^, the mechanisms that regulate early events of adipocyte differentiation have not been completely defined^11^. Consequently, understanding the mechanisms regulating adipogenesis should provide valuable information for the development of new anti-obesity therapies.

One of the endogenous factors proposed to regulate adipocyte differentiation is HMGA1 (High Mobility Group AT-hook-1). HMGA1 belongs to a large family of architectural nuclear proteins that bind to DNA and nucleosomes and regulate diverse cellular processes by inducing changes in the chromatin fiber^12^,^13^. During embryonic development *Hmga1* expression is gradually down-regulated, suggesting that proper development and differentiation requires regulated levels of HMGA1^13^,^14^. In agreement with this, failure to down-regulate *Hmga1* reprograms somatic cells to an undifferentiated/stem cell-like state^15^,^16^. Similarly, HMGA1 is differentially expressed during adipogenesis *in vitro*, being expressed early in undifferentiated 3T3-L1 preadipocytes and decreasing during adipocyte maturation, suggesting that HMGA1 may exert a negative effect on adipogenesis and that downregulation of HMGA1 expression may be necessary for adipocyte terminal differentiation *in vitro*^17^. However, the exact role of *Hmga1* in adipogenesis and adipose tissue functionality *in vivo* remains unknown.

To evaluate the physiological role of HMGA1 in adipogenesis *in vivo* and its potential pathophysiological effects during obesity, we created transgenic mice overexpressing HMGA1 in adipose tissue. HMGA1 transgenic mice presented impaired terminal differentiation of white and brown adipocytes and a marked reduction in body fat that did not lead to lipodystrophic diabetes; instead, HMGA1 transgenic mice showed not only reduced body weight gain, but also improved glucose tolerance and insulin sensitivity after diet-induced obesity. Our results suggest that HMGA1 plays an important function in the regulation of white and brown adipogenesis *in vivo* and protects against obesity and related metabolic diseases.

## Results

### Adipose overexpression of HMGA1 led to reduced adiposity

**Figure 1:**
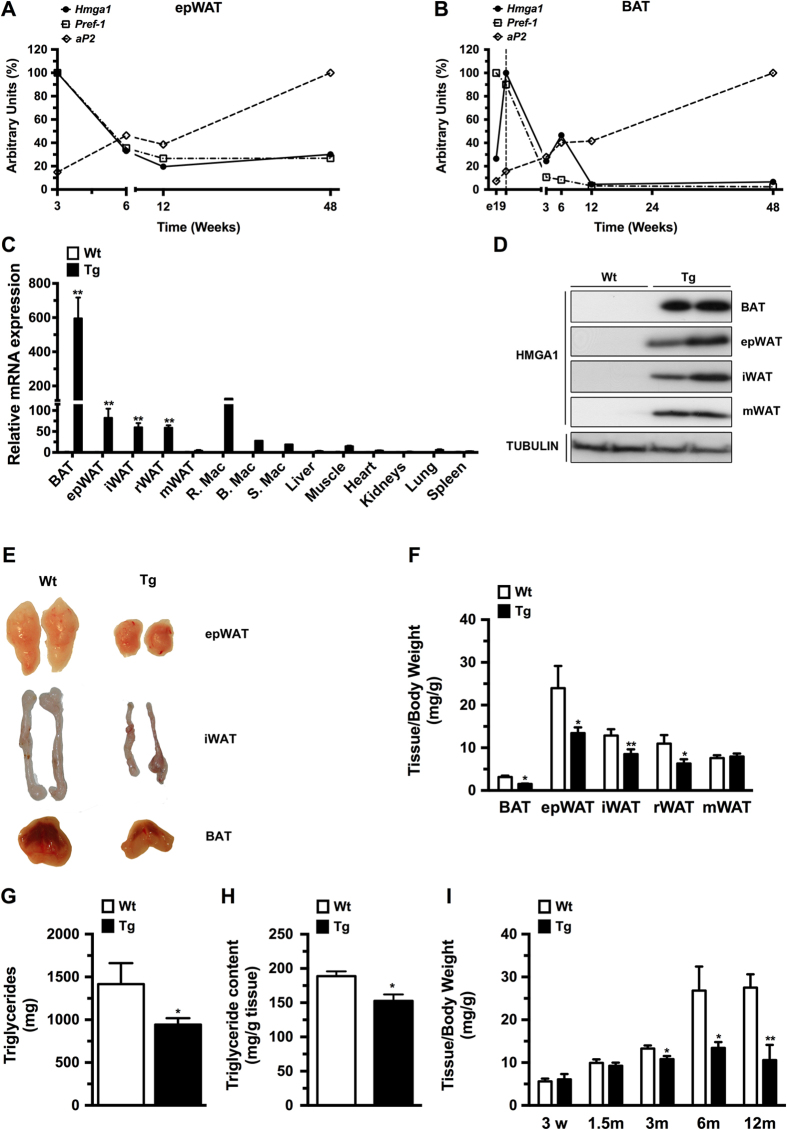
Adipose-specific overexpression of HMGA1 led to lower adiposity. (**A**) Post-natal HMGA1 expression in epWAT from wild-type mice. (**B**) Follow-up of HMGA1 expression in BAT from wild-type mice. (**C**) HMGA1 mRNA levels in adipose and non-adipose tissues in 6-month-old mice (R.Mac.: Resident epididymal WAT (epWAT) macrophages; B.Mac.: Blood macrophages; S.Mac.: Spleen Macrophages). (**D**) Representative Western blots from different fat depots probed with an antibody against HMGA1 and showing a band of approximately 11.5 KDa corresponding to HMGA1. Full-length blots are presented in Supplementary Figure 8. (**E**) Representative macroscopic images of different fat pads (epididymal WAT (epWAT); inguinal WAT (iWAT), and BAT). (**F**) Weight of BAT and different white fat depots (epididymal WAT (epWAT); inguinal WAT (iWAT); retroperitoneal WAT (rWAT); and mesenteric WAT (mWAT)) normalized to total body weight (*n* = six mice/group). (**G**) Total triglyceride content in whole body carcasses (*n* = six mice/group). (**H**) Specific triglyceride content in epWAT (*n* = six mice/group). (**I**) Changes in epWAT fat depot weight normalized to body weight (*n* = eight mice/group). Data are means ± SEM. ^*^P ≤ 0.05 and ^**^P ≤ 0.01 *vs.* wild-type mice.

Firstly, to determine whether HMGA1 was associated with the development of adipose tissue, we analyzed *Hmga1* gene expression at distinct stages of normal post-natal development in mouse white (WAT) and brown (BAT) adipose tissues. In accordance with the developmentally regulated expression of *Hmga1*^14^, *Hmga1* was highly expressed in WAT and BAT at early stages of development and gradually decreased during adulthood until 12 weeks where it reached a plateau (Fig. 1A,B). Interestingly, *Hmga1* expression paralleled that of preadipocyte factor-1 (*Pref-*1), a preadipocyte marker gene^2^,^10^, and was opposite to that of the mature adipocyte marker fatty acid binding protein (*Fabp4*), also known as adipocyte protein 2 (*aP2*) (Fig. 1A,B)^2^,^10^, suggesting that HMGA1 might play an important role in the early stages of WAT and BAT development *in vivo*.

Next, to evaluate the role of HMGA1 in adipogenesis and adipocyte function *in vivo*, we produced transgenic mice that overexpressed HMGA1 from the *aP2* promoter. The aP2 promoter is mostly used to drive specific expression of transgenes to the adipose tissue^18^. However, it has been described that the aP2 protein is also expressed in a lesser extent in other tissues^19^,^20^,^21^. As expected, aP2-HMGA1 transgenic mice showed a marked increase in *Hmga*1 mRNA levels in adipose tissues and macrophages compared to wild-type mice (Fig. 1C). Expression of Hmga1 was also detected in non-adipose tissues of transgenic mice, although to a much lesser extent than the observed in adipose depots (Fig. 1C), an expression that could be attributable to the resident macrophages present in these tissues^21^. Protein levels were also highly increased in all WAT depots (epididymal (epWAT), mesenteric (mWAT) and inguinal-subcutaneous (iWAT)) and in interscapular BAT (BAT) of transgenic mice (Fig. 1D). The weight of all WAT and BAT depots, except mWAT, was significantly reduced in transgenic mice (Fig. 1E,F and Supplementary Figure 1A), which was related to a pronounced decrease in whole-body triglyceride content (Fig. 1G). No significant differences were observed in body weight (Wild-type = 33.2 ± 0.7 g *vs*. Transgenic = 35.5 ± 1.2 g) or in the weight of non-adipose tissues (Supplementary Figures 1B and 1C). The reduction of epWAT weight paralleled a decrease in both the size of adipocytes (mean surface area: Wild-type = 3201 ± 40 μm^2^ *vs.* Transgenic = 3053 ± 40 μm^2^; *P *< 0.01) and in their triglyceride content (Fig. 1H). The reduced weight of the epWAT depot in aP2-HMGA1 mice compared to wild-type became significantly different at 3 months of age, but was even more pronounced at 6 and 12 months of age (Fig. 1I). Collectively, these results indicated that adipose-specific HMGA1 overexpression led to partial lipodystrophy in aP2-HMGA1 transgenic mice.

### aP2-HMGA1 mice presented normal glucose tolerance and insulin sensitivity

**Figure 2:**
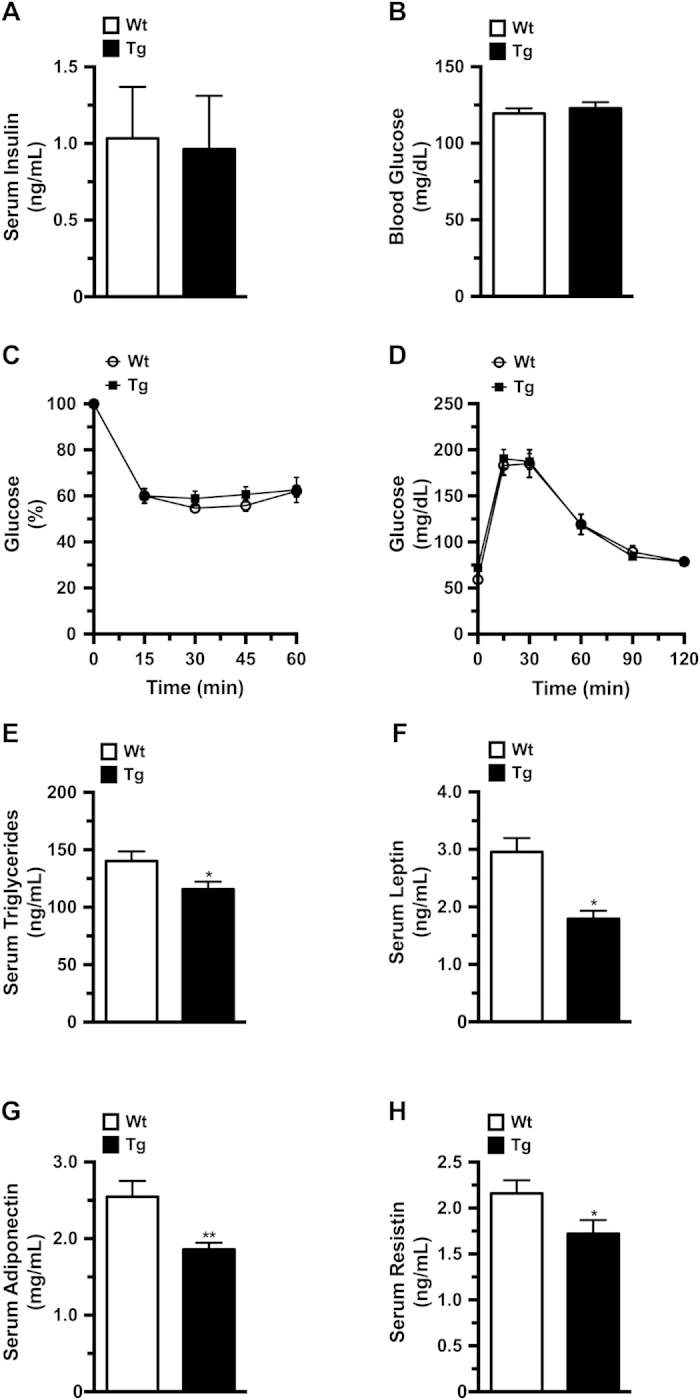
aP2-HMGA1 mice presented normal glucose tolerance and insulin sensitivity. Levels of circulating metabolites were enzymatically determined. (**A**) Serum insulin levels (*n* = six mice/group) and (**B**) Blood glucose levels (*n* = six mice/group). (**C**) Insulin sensitivity was determined after an intraperitoneal injection of insulin (0.75 IU/kg body weight). Results are calculated as the percentage of initial blood glucose levels (*n* = ten mice/group). (**D**) Glucose tolerance was determined in fasted mice after an intraperitoneal injection of glucose (1 g/Kg body weight), and blood glucose levels were measured at the indicated time points (*n* = ten mice/group). (**E**) Levels of serum triglycerides (*n* = six mice/group). (**F**) Levels of serum leptin (*n* = six mice/group). (**G**) Levels of serum adiponectin (*n* = six mice/group). (**H**) Levels of serum resistin (*n* = six mice/group). Values shown are means ± SEM. ^*^P ≤ 0.05 and ^**^P ≤ 0.01 *vs.* wild-type mice.

Despite lower adipose tissue mass, aP2-HMGA1 mice did not show the metabolic consequences observed in lipodystrophy. Indeed, aP2-HMGA1 mice did not show alterations in serum insulin and glucose levels (Fig. 2A,B) and exhibited similar insulin sensitivity and glucose tolerance to wild-type mice at 3, 6 or 12 months of age (Fig. 2C,D and Supplementary Figure 2). However, decreased serum levels of triglycerides as well as leptin, adiponectin and resistin were detected in aP2-HMGA1 mice (Fig. 2E–H) and accumulation of triglycerides was not observed in the liver or skeletal muscle of transgenic mice at 3, 6 or 12 months of age (not shown).

### aP2-HMGA1 transgenic mice showed altered adipogenesis in WAT

**Figure 3:**
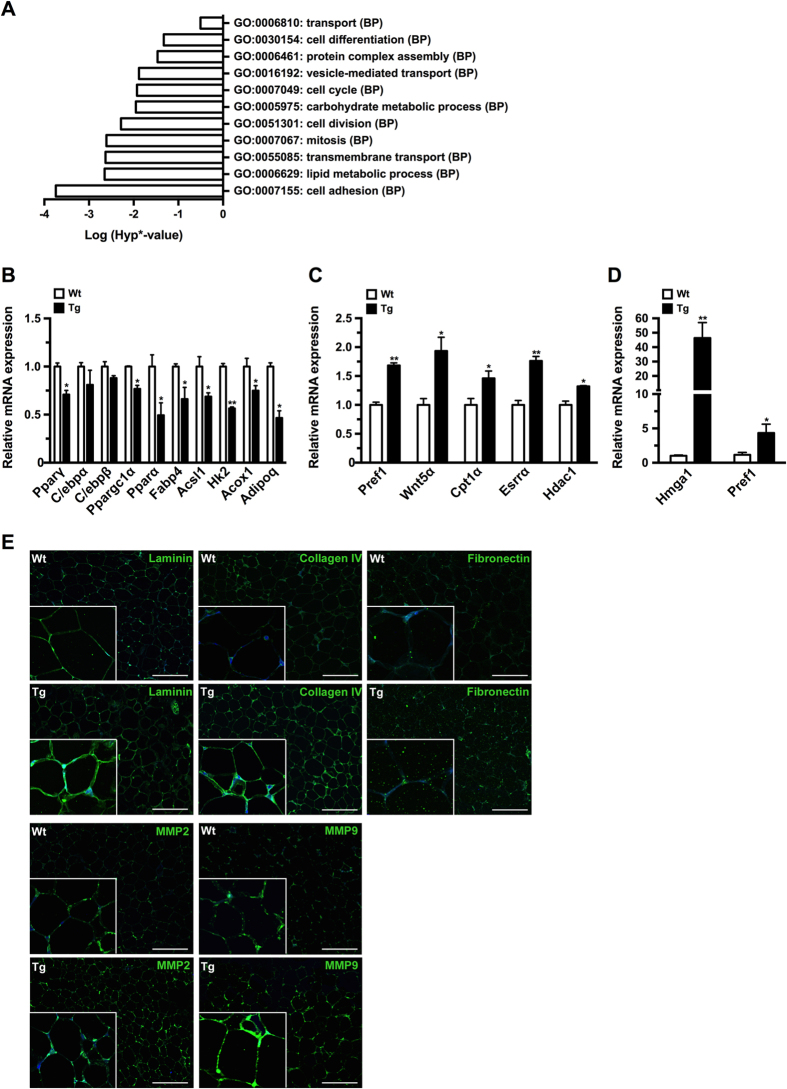
aP2-HMGA1transgenic mice showed altered adipogenesis in epWAT. (**A**) Dysregulated gene ontology (GO) biological processes (BPs) in epWAT from aP2-HMGA1 in 6-month old mice (*n* = three). (**B**) Downregulated genes in epWAT from transgenic mice (*n* = four mice/group). (**C**) Upregulated genes in epWAT from transgenic mice (*n* = four mice/group). (**D**) Increased preadipocyte marker expression in the SVF of aP2-HMGA1 transgenic mice. *Hmga1, Pref-*1 expression levels in the SVF of epWAT (*n* = three tissue pools of three mice/pool). (**E**) Representative immunostaining against laminin, collagen IV, fibronectin, MMP2, and MMP9 (green) in sections of epWAT. Nuclei (blue). Original magnification X200 and X400 (*insets*). Scale bars: 51.31 μm. Values shown are means ± SEM. ^*^P ≤ 0.05 and ^**^P ≤ 0.01 *vs.* wild-type mice.

To identify the genes and cellular processes regulated by HMGA1 in adipocytes *in vivo*, we used microarrays to compare the expression profile of epWAT of wild-type and transgenic mice. After array processing, Gene Ontology (GO) analysis showed aberrant regulation of several processes involved in adipogenesis and WAT biology in transgenic mice, such as cell differentiation, cell cycle, cell division, and carbohydrate and lipid metabolic processes (Fig. 3A). From the list of aberrant processes we selected a number of marker genes putatively related to adipogenesis to both confirm the array data and to further explore the underlying molecular processes. Real-time quantitative PCR (qPCR) analysis revealed a marked downregulation in the expression of pro-adipogenic transcription factors, such as *Ppar*γ, and adipocyte metabolic genes, like *Ppar*α, *Fabp*4 and adiponectin (*Adipoq*) in transgenic mice (Fig. 3B). Conversely, the expression of differentiation repressor genes, such as *Pref-*1 and Wingless-type-5a (*Wnt5a*) was upregulated in epWAT of aP2-HMGA1 mice (Fig. 3C), suggesting that expression of HMGA1 may inhibit adipogenesis *in vivo*. Next, we isolated WAT stromal vascular fraction (SVF) from wild-type and transgenic mice to investigate whether HMGA1 activation changed the fate of adipose progenitors. qPCR analysis showed higher levels of *Pref-*1 in the SVF of aP2-HMGA1 mice compared to wild-type (Fig. 3D and Supplementary Figure 3), which suggested that HMGA1 may block adipogenesis by increasing adipocyte precursors in the SVF of adipose tissues. Moreover, up-regulation of extracellular matrix (ECM) and tissue remodelling components, such as laminin, collagen IV or matrix metalloproteinase-9 (MMP9), was detected in epWAT of transgenic mice (Fig. 3E). Similar ECM alterations were observed in other mouse models in which adipogenesis has been disrupted^22^,^23^, further supporting the impairment of adipogenesis observed in epWAT of aP2-HMGA1 mice. Similar results were observed when we analyzed gene expression and triglyceride content in iWAT from transgenic mice (Supplementary Figure 4). Taken together, these results suggested that adipose specific expression of HMGA1 in WAT impairs adipocyte differentiation *in vivo*.

### HMGA1 overexpression impaired BAT development

**Figure 4:**
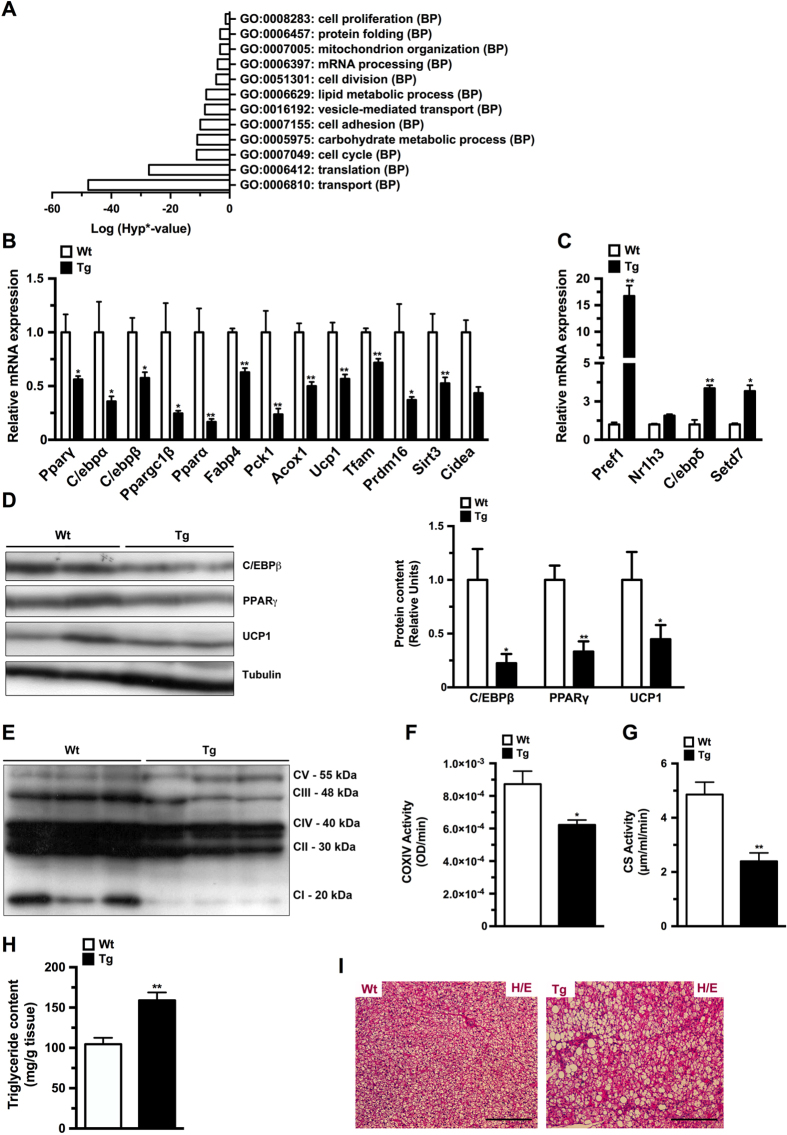
HMGA1 overexpression impaired BAT development and function. (**A**) Dysregulated biological process (BP) gene ontologies (GO) categories in BAT from aP2-HMGA1 transgenic mice (*n* = three mice/group). (**B**) Downregulated genes in BAT from transgenic mice (*n* = four mice/group). (**C**) Upregulated genes in BAT from transgenic mice (*n* = four mice/group). (**D**) Representative Westerns blots (WB) of adipogenic transcription factors in BAT and relative quantification of protein expression. Full-length blots are presented in Supplementary Figure 8. (**E**) Representative WB of mitochondrial OXPHOS complex proteins. Full-length blot is presented in Supplementary Figure 9. (**F**) Enzymatic cytochrome oxidase (COX) IV activity (*n* = five mice/group). (**G**) Enzymatic citrate synthase activity (*n* = five mice/group). (**H**) Specific triglyceride content in BAT (*n* = five mice/group). (**I**) Representative sections of BAT stained with hematoxylin/eosin (original magnification X100). Data are mean ± SEM. ^*^P ≤ 0.05 and ^**^P ≤ 0.01 *vs.* wild-type mice.

aP2-HMGA1 transgenic mice showed a marked decrease in BAT (Fig. 1E,F), suggesting that development of BAT was also impaired. Similar to our previous observations in epWAT microarray analysis of BAT gene expression in aP2-HMGA1 mice, showed alterations in genes associated with differentiation processes (cell differentiation, cell division, cell cycle) and metabolism (mitochondrion organization, lipid and carbohydrate metabolic processes) (Fig. 4A). qPCR analysis confirmed the marked downregulation in the expression of adipocyte differentiation genes (such as *Pparγ*, *C/ebpα* and PR domain containing-16 (*Prdm16*)) as well as adipocyte metabolism genes, such as *Pparα*, uncoupling protein-1 (*Ucp1)* and mitochondrial transcription factor-A (*Tfam*) in BAT of aP2-HMGA1 mice (Fig. 4B). Conversely, the expression of adipocyte precursors and differentiation repressor genes, like *Pref*-1, were upregulated in BAT of transgenic mice (Fig. 4C). Moreover, a marked increase in BAT remodelling processes was detected in aP2-HMGA1 transgenic mice (Supplementary Figure 5A), thus suggesting that HMGA1 also inhibits adipogenesis in BAT development.

Furthermore, in accordance to the gene expression data, aP2-HMGA1 transgenic mice had lower protein levels of key transcription factors involved in BAT adipogenesis (such as *C/ebpβ* and *Pparγ*; Fig. 4D), which was parallel to decreased expression of proteins involved in BAT mitochondrial function, such as UCP1 (Fig. 4D) and the OXPHOS protein complex (Fig. 4E). In agreement with the decreased expression of mitochondrial genes and proteins observed in transgenic mice, complex IV (Fig. 4F) and citrate synthase (Fig. 4G) enzymatic activities were reduced in BAT from transgenic mice. In addition, downregulation in the expression of carnitine palmitoyltransferase 1B (*Cpt1b*), a key enzyme in fatty acid oxidation, as well as in the expression of hormone sensitive lipase (*Lipe*) and Patatin-like phospholipase domain-containing protein 3 (*Pnpla3*) was detected in transgenic mice (Supplementary Figure 5B), which suggested a decrease in BAT lipolytic activity of transgenic mice. As a result, aP2-HMGA1 mice showed increased triglyceride accumulation in BAT (Fig. 4H) and brown adipocytes exhibited large unilocular triacylglycerol-filled vacuoles (Fig. 4I). A similar enlargement of lipid droplets is observed in other mouse models in which the development or function of BAT has been impaired^24^,^25^. Taken together, these data indicated that HMGA1 also blocked the development and function of BAT.

### HMGA1 overexpression prevented high fat diet-induced obesity

**Figure 5:**
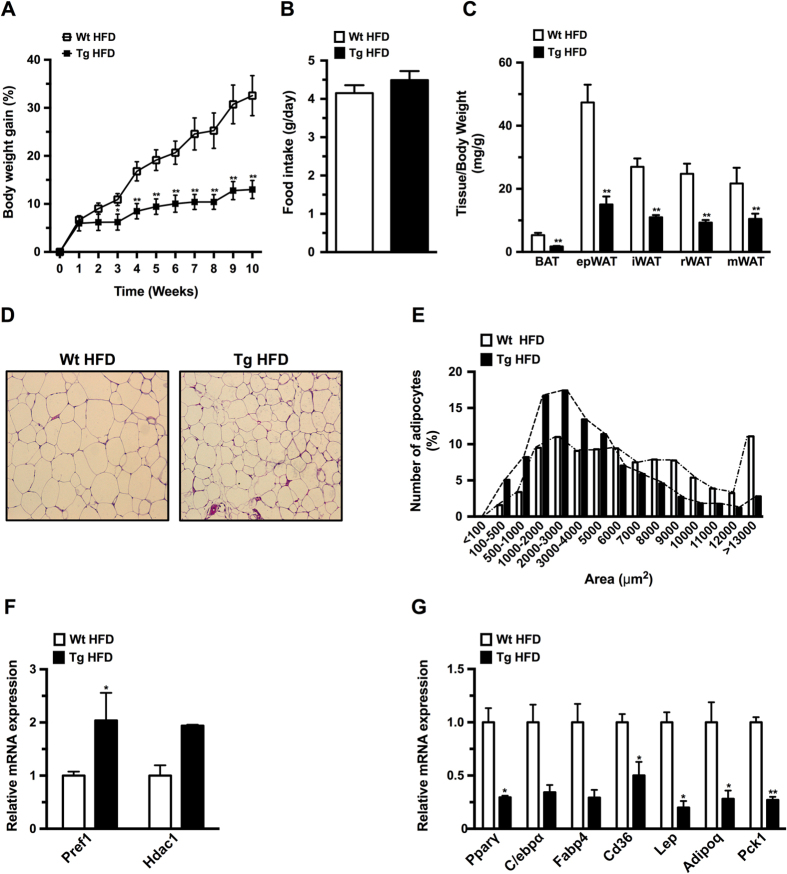
HMGA1 inhibited development of high fat diet-induced obesity. (**A**) Body weight gain (%) was determined in both wild-type (Wt) and aP2-HMGA1 transgenic mice (Tg) fed high-fat diet (HFD) for 10 weeks (*n* = sixteen mice/group). 12 week-old mice were placed into the HFD. (**B**) Food intake of wild-type (Wt) and transgenic mice (Tg) fed high-fat diet was measured as daily food intake per mouse (g/day). (**C**) Adipose tissue depots weight was measured and normalized to body weight (*n* = seven mice/group). (**D**) Representative hematoxylin/eosin stained sections of epididymal WAT (epwAT) (original magnification X100). (**E**) Frequency distribution of adipocyte area in epWAT (*n* = six mice/group). (**F**) Upregulated genes in epWAT from transgenic mice (*n* = four mice/group). (**G**) Downregulated genes in epWAT from transgenic mice fed a HFD (*n* = four mice/group). Data are mean ± SEM. ^*^P ≤ 0.05 and ^**^P ≤ 0.01 *vs.* wild-type fed a HFD mice.

To determine the impact of impaired adipogenesis on the development of obesity we fed wild-type and transgenic mice a high fat diet (HFD). Ten weeks after commencing a HFD, aP2-HMGA1 transgenic mice showed a marked decrease in body weight gain compared to wild-type mice (Fig. 5A and Supplementary 6A), which was not due to differences in food intake (Fig. 5B and Supplementary Figure 6B), indicating that transgenic mice were more energetically inefficient than wild-type littermates (Supplementary Figure 6C). Consistent with what we observed under a STD-chow diet, the weight of WAT and BAT depots was markedly reduced in aP2-HMGA1 mice fed a HFD (Fig. 5C and Supplementary Figure 6D). No differences in the weight of other tissues were observed, except for an increase in liver weight in transgenic mice (Supplementary Figures 6E and 6F), which was related to increased triglyceride deposition (Wild-type = 55 ± 8 mg/mL *vs.* Transgenic = 98 ± 8 mg/mL; *P* < 0.05).

White adipocytes from HFD-fed transgenic mice were smaller (Fig. 5D), with an increased frequency of small and intermediate-sized adipocytes, as well as reduced number of large adipocytes, compared to wild-type mice (Fig. 5E). Similar to STD-fed mice, elevated levels of *Pref-*1, as well as reduced levels of other adipogenic and metabolic genes were also detected in the epWAT of HFD-fed transgenic mice (Fig. 5F,*G*). Alterations in the frequency distribution of adipocyte size as well as impaired gene expression were also observed in iWAT (Supplementary Figures 6G and 6H) and BAT (Supplementary Figure 6I) of HFD-fed aP2-HMGA1 mice. Collectively, these data suggested that HMGA1 overexpression protected transgenic mice from diet-induced obesity, likely due to the decreased adipocyte differentiation capacity in WAT and BAT depots.

### aP2-HMGA1 mice were protected against diet-induced insulin resistance

**Figure 6:**
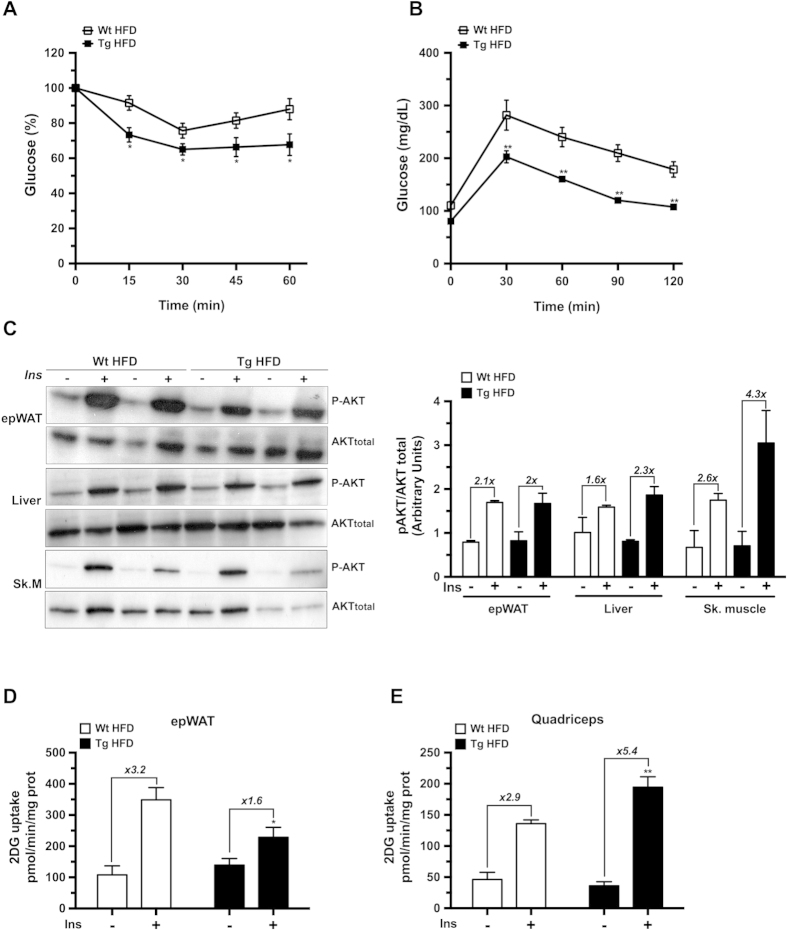
aP2-HMGA1 transgenic (Tg) mice were protected against diet-induced insulin resistance. (**A**) Insulin sensitivity was determined after an intraperitoneal injection of insulin (0.75 U/Kg body weight). Results are calculated as the percentage of initial blood glucose levels (*n* = nine mice/group). (**B**) Glucose tolerance was determined after an intraperitoneal injection of glucose (1 g/Kg), and blood glucose levels were measured at the indicated time points (*n* = nine mice/group). (**C**) Representative Western blots are shown for phosphorylated Akt (p-AKT) and total Akt (AKT total) before and after insulin stimulation in epWAT, liver and skeletal muscle, as indicated in METHODS and relative quantification of protein expression (*n* = four mice/group). Full-length blots are presented in Supplementary Figure 10. (**D**) *In vivo* 2-DG-glucose uptake by epWAT from mice fed a HFD (*n* = five mice/group). (**E**) *In vivo* 2-DG-glucose uptake by skeletal muscle (quadriceps) from mice fed a HFD (*n* = five mice/group). Data are means ± SEM. ^*^P ≤ 0.05 and ^**^P ≤ 0.01 *vs.* wild-type mice fed a HFD.

Despite impaired WAT differentiation and a lower triglyceride storage capacity, and a reduced BAT oxidative capacity, HFD-fed aP2-HMGA1 mice were more insulin sensitive and glucose tolerant than wild-type mice (Fig. 6A,B). No differences in basal levels of glucose and insulin basal levels were observed between HFD-fed transgenic and wild-type mice (Table 1). However, aP2-HMGA1 mice showed reduced serum levels of lipid metabolites and adipokines compared to wild-type mice (Table 1), further suggesting that aP2-HMGA1 mice were protected against diet-induced insulin resistance. To further characterize the protection against diet-induced insulin resistance observed in aP2-HMGA1 mice, we analyzed the insulin-stimulated phosphorylation of Akt (p-Akt) as well as 2-deoxyglucose (2-DG) uptake in WAT, liver and skeletal muscle in these mice. Upon insulin stimulation, p-Akt levels were unchanged in both WAT and liver (Fig. 6C). However, decreased 2-DG uptake was observed in WAT of aP2-HMGA1 HFD-fed mice (Fig. 6D and Supplementary Figure 7A). Interestingly, increased p-Akt levels were observed in skeletal muscle from insulin-stimulated transgenic mice compared to wild-type mice (Fig. 6C). Furthermore, insulin-stimulated 2-DG uptake was also markedly increased in skeletal muscle from HFD-fed aP2-HMGA1 mice (Fig. 6E and Supplementary Figure 7B). The expression of genes involved in insulin signalling, lipid catabolism and glucose metabolism were upregulated in skeletal muscle of aP2-HMGA1 mice (Supplementary Figure 7C). Moreover, glycogen accumulation was increased in the skeletal muscle of HFD-fed transgenic mice compared to wild-type mice (Wild-type = 1.7 ± 0.3 mg/kg *vs.* Transgenic = 2.35 ± 0.5 mg/kg) further suggesting increased insulin sensitivity. Taken together, these results suggested that skeletal muscle might largely contribute to the amelioration of whole-body glucose homeostasis and insulin resistance observed in HFD-fed aP2-HMGA1 transgenic mice, despite the lipodystrophy exhibited by these mice.

## Discussion

To determine the role of HMGA1 during adipose tissue development and its implications in obesity, we created aP2-HMGA1 mice. In these transgenic mice, overexpression of HMGA1 impaired both white and brown adipose tissue development and led to lower adiposity than wild-type mice. Our results are in agreement with previous observations in other mouse models where the wild-type form of HMGA1 was overexpressed ubiquitously and which did not accumulate fat^26^. In contrast, transgenic mice ubiquitously expressing a truncated HMGA1 gene accumulated abundant ectopic fat^27^. However, in both studies, the authors did not analyze adipose tissue alterations and focused on the effects of HMGA1 expression in tumours of the lymphoid system^26^,^27^. Although several studies have linked HMGA1 to oncogenesis^13^,^28^, HMGA1 might be both pro- and anti-oncogenic depending on the cellular context^29^. In this regard, we have not observed a higher incidence of tumours in aP2-HMGA1 mice.

Impaired terminal differentiation of adipocytes has been claimed to be responsible for lower fat mass in both WAT and BAT^25^,^30^,^31^. Gene expression analysis of WAT and BAT from aP2-HMGA1 transgenic mice showed downregulation of the genes involved in adipocyte differentiation and upregulation of preadipocyte marker genes. In addition, the natural expression of *Hmga*1 in adipocytes from wild-type mice followed the same temporal pattern as the preadipocyte marker gene *Pref*-1 in WAT and BAT^32^,^33^, as both genes decreased their expression levels as adipocyte differentiation progressed. Accordingly, the phenotype of aP2-HMGA1 mice was reminiscent of that of mice overexpressing *Pref-*1 in WAT^34^,^35^. Therefore, downregulation of both *Pref-*1 and *Hmga*1 expression may be necessary for complete adipocyte terminal differentiation. Similarly, overexpression of HMGA1 has also been shown to prevent terminal differentiation of myocytes, keeping them in an undifferentiated state due to specific alterations in chromatin remodelling that interfere with the expression of myogenic genes^16^. Likewise, elevated levels of chromatin remodelling proteins involved in the inhibition of adipogenesis, such as histone deacetylase-1 (*Hdac*1)^36^, were observed in adipose tissue of aP2-HMGA1 mice, further suggesting the maintenance of undifferentiated/preadipocytic state in the adipose depots of transgenic mice. HMGA1 signalling has also been reported to reprogram somatic cells into an undifferentiated/stem cell-like state^15^. In this regard, aP2 expression has been described to mark a population of undifferentiated adipocyte progenitors in WAT and BAT^37^, and thus may drive *Hmga*1 expression in the progenitor pool. Therefore, our hypothesis is that aP2-driven *Hmga*1 expression, together with the elevated expression of *Pref-*1 and other preadipocyte markers, probably leads to an increase in adipose precursor pool population, maintaining the undifferentiated state after preadipocyte commitment, but prior to adipocyte differentiation. Further experiments will be carried out to characterize the mechanisms underlying the commitment of aP2-HMGA1 preadipocytes.

Perhaps the most surprising results of overexpressing HMGA1 within adipose tissue were the anomalous phenotypes regarding glucose homeostasis that seem paradoxical to reports on classical lipodystrophy^4^,^5^. aP2-HMGA1 mice presented a marked decrease in fat depot mass, but did not show the metabolic consequences related with lipodystrophy and were protected not only against diet-induced obesity, but also against systemic insulin resistance. Therefore, impaired adipocyte differentiation and decreased fat mass is not always associated with impaired whole body glucose homeostasis. Similarly, genetically engineered mice with impaired development of adipose tissue as a result of altered COUP-TF II^38^, TFAM^39^ or Wnt signalling pathways^25^,^30^,^31^ also showed reduced weight gain and normalization of glucose and insulin metabolism. Furthermore, retinol-binding protein 4 (RBP4) has been described to contribute to insulin resistance in obesity and type 2 diabetic patients^40^,^41^. Chiefari *et al.*, using the hmga1 knock-out mice model, have reported that HMGA1 is needed for the expression of RBP4 by mechanisms that involve cAMP^42^. However, we have not observed any differences in the expression of RBP4 mRNA in the microarray experiment, in which we analyzed the gene expression profile of WAT and BAT from wild-type and transgenic mice. In spite of our observations, we cannot rule out the possibility that RBP4 might be involved in the regulation of the adipocyte differentiation and glucose homeostasis in HMGA1 transgenic mice. Therefore, further experiments are needed to elucidate the downstream effects of HMGA1.

Intriguingly, increased skeletal muscle glucose uptake appeared to be an important catalyst of the improved glucose homeostasis and insulin sensitivity in aP2-HMGA1 mice. Similarly, transgenic mice with activated Wnt signalling in adipose progenitors showed impairment of adipogenesis, but increased muscle glucose uptake, which led to improvements in glucose homeostasis^31^. How altered adipogenesis, as a result of HMGA1 overexpression specifically in adipocytes, affects glucose metabolism in muscle is unclear. Therefore, further experiments would also be needed in order to characterize the mechanisms underlying the crosstalk between adipose tissue and skeletal muscle in aP2-HMGA1 transgenic mice.

Interestingly, several genetic studies in human populations have revealed a close association between insulin resistance and type 2 diabetes with polymorphic variants in the gene encoding for HMGA1^43^,^44^,^45^,^46^. Moreover, it has been reported that patients deficient in HMGA1 exhibit insulin resistance and type 2 diabetes^47^. However, no associations have been reported between HMGA1 polymorphisms and body weight in these studies. Nevertheless, our results in aP2-HMGA1 transgenic mice, together with the aforementioned human studies, support the notion that HMGA1 is a key player in the maintenance of glucose homeostasis.

In summary, the adipocyte-specific overexpression of HMGA1 resulted in impaired development of white and brown adipose tissues through the inhibition of the adipogenic process, resulting in increased adipose precursor pool populations. Moreover, aP2-HMGA1 transgenic mice were protected against diet-induced obesity and its metabolic complications, providing evidence that impaired adipocyte differentiation and decreased fat mass is not always associated to impaired whole body glucose homeostasis. Therefore, HMGA1 appears as a plausible new therapeutic target against obesity and related metabolic disorders.

## Methods

### Animals

Transgenic mice that express mouse HMGA1 cDNA under the control of the 5.4-kb mouse aP2 promoter were created by pronuclear injection into C57Bl/6 mice in the transgenic core facility at the CBATEG-UAB. Similar expression patterns and phenotypes were observed in two founder lines; however, studies reported herein are from a single transgenic line. Mice were maintained under a 12-h light–dark cycle at 22 °C in a pathogen-free facility. Unless otherwise stated, male mice aged 3–6 months were used. Mice were fed *ad libitum* with a chow diet (2018S Harlan Teklad, USA) or a high fat diet (HFD) (TD88137 Harlan Teklad). When stated, mice were fasted for 16 h.

### Ethics statement

All experimental procedures were approved by the Ethics and Experimental Animal Committee of the Universitat Autònoma de Barcelona (UAB). Methods were carried out in accordance with the approved guidelines of the Universitat Autònoma de Barcelona (UAB).

### Isolation of stromal vascular fraction (SVF)

To isolate adipocytes and the SVF from BAT and epididymal white adipose tissue (epWAT), depots were collected and digested for 20–30 min at 37 °C with collagenase A (2 mg/ml) in Dulbecco’s Modified Eagle’s Medium (DMEM) containing 2% bovine serum albumin. The resulting cell suspension was filtered through a nylon 100 μm strainer and left to stand for 20 min to allow flotation of adipocytes. Floating adipocytes were collected with a pipette while the remaining digestion solution was centrifuged at 500 g for 10 min to collect SVF cells^24^. SVF was immediately processed for RNA isolation.

### Flow cytometry

Macrophages were isolated from spleen, blood and epWAT SVF of wild-type and transgenic mice and incubated with specific antibodies for sorting: phycoerythrin (PE) anti-mouse cluster of differentiation (CD)11b (BD Pharmigen 557397, Franklin Lakes, NJ), PE/Cy7 anti-mouse CD11b (Biolegend 101216, San Diego, CA), PE anti-mouse CD11c (BD Pharmigen 557401) and fluorescein isothiocyanate anti-mouse F4/80 (Biolegend 123107). Sorting analysis was performed with a BD FacsAria III cell sorter (BD Biosciences) equipped with four lasers (488 nm, 633 nm, 405 nm and 561 nm). Data were analysed using BD FACSDiva^TM^ v6.2 software.

### Gene expression analysis

For quantitative real-time PCR analysis, total RNA was first extracted from most tissues with Trizol® (Invitrogen, USA) or from fat tissues with QIAZOL (Qiagen, Germany), and then with a RNeasy Mini Kit (Qiagen). Total RNA (1 μg) was retrotranscribed using the Transcriptor First Strand cDNA Synthesis Kit (Roche Diagnostics, Switzerland). Quantitative PCR was performed in a LightCycler480 II (Roche) using the Light Cycler probe for Real Time ready Custom Panel 384–32 (Roche). Relative expression was calculated according to the Pfaff I method^48^. Data were normalized calculating the geometric mean of preplatted *Rplp0*, *Hprt* and *Gapdh* used as references genes.

For microarray analyses, biotinylated complementary (cRNA) was synthesized from 300 ng of total RNA using the GeneChip 3′ IVT Express kit (Affymetrix, USA) following the manufacturer’s recommendations. Affymetrix gene chips (Mouse Genome 430 2.0A arrays, Affymetrix) were used for hybridization and data collection. The protocol was performed by the Microarray Facility at Progenika (Bilbao, Spain). The microarray data were processed with the AffymetrixGeneChip Command Console Software (AGCC 2.0, Affymetrix) and Expression Console™ (EC 1.1, Affymetrix). The final gene list contained only those probe-sets with a *P* < 0.05. Functional analysis was done using the online tool David^49^. For statistical analysis, we considered Hyp*-value as statistically significant^48^.

### Western blot

Tissues were homogenized in protein lysis buffer as previously described^50^. All gels were run under the same experimental conditions. Primary antibodies were anti-HMGA1a/HMGA1b (ab4078, Abcam, UK); anti-C/EBPβ (C-19) (sc-150, Santa Cruz, USA); anti-PPARγ (ab5907, Abcam); anti-UCP1 (ab10983, Abcam); anti-MitoProfile®total OXPHOS Rodent WB antibody cocktail (ab110413, Abcam); anti-Akt (#9272, Cell Signaling, USA) and anti-phospho-Akt (Ser473) (#9271, Cell Signaling). Image processing was performed using ImageJ 1.48v free online software.

### BAT Citrate synthase (CS) and Complex IV enzyme activities

CS activity was measured as described by Pardo *et al.*^51^. Mitochondrial cytochrome C oxidase (complex IV) activity was measured in BAT homogenates using a Complex IV Rodent Enzyme Activity Microplate Assay Kit, following the manufacturer’s recommendations (Abcam).

### Metabolite and hormone assays

Triglyceride content in mouse carcasses was analyzed as previously described^52^. Tissue triglyceride content was determined by extracting total lipids with chloroform-methanol (2:1 vol/vol), as described^53^. Triglycerides were quantified spectrophotometrically using an enzymatic assay kit (Horiba Medical-ABX, France). Serum free fatty acids (FFAs) were measured by the acyl-CoA synthase and acyl-CoA oxidase methods (Wako Chemicals GmbH, Germany). Serum glycerol concentration was determined enzymatically (Randox Laboratory, UK). All biochemical parameters were determined using a Pentra 400 Analyzer (Horiba Medical-ABX). Glucose was determined using a Glucometer Elite analyzer (Bayer, Germany), and insulin levels were measured using the Sensitive Rouse Insulin RIA Kit (Millipore, USA). Leptin and resistin were measured using the Milliplex Mouse Adipokine Panel (MADPK-71K, Merck Millipore, Germany) using Millipore reagents and instructions. Adiponectin concentration was determined by RIA using the Mouse Adiponectin RIA Kit (MADP-60HK, Merk Millipore).

### Histological, immunohistochemical and morphometric analysis

Tissues were fixed for 24 h in formalin, embedded in paraffin and sectioned. For immunohistochemical detection, sections were deparaffinised and incubated overnight at 4 °C with specific antibodies: polyclonal rabbit anti-laminin (Z0097, DakoCytomation); polyclonal antibody anti-collagen, type IV (AB756P, Merck-Millipore); anti-human fibronectin (F3648, Sigma); rabbit polyclonal anti-matrix metalloproteinase-2 (MMP2) (ab79781, Abcam); rabbit polyclonal anti-MMP9 (ab38898, Abcam); washed with PBS and incubated with the corresponding secondary antibodies and developed with ABC Complex (Vector Laboratories Ltd., UK). Morphometric analysis of adipocyte size was performed in epWAT sections stained with hematoxylin-eosin. Adipocyte area was determined as previously described^54^. Five animals per group were used and at least 400 adipocytes per animal were analyzed.

### Glucose and insulin tolerance tests

Mice were given an intraperitoneal injection of glucose (1 g/Kg of body weight) or insulin (0.75 IU/Kg body weight) and the glucose concentration was determined in blood samples at the indicated time points.

### Insulin signalling

Overnight-fasted animals were anaesthetized and, after being clamped, a portion of epWAT, quadriceps, gastrocnemius and liver were excised and frozen in liquid nitrogen. Immediately, mice were injected with an intraperitoneal injection of insulin (5 IU/g body weight). Fifteen minutes after insulin stimulation, the remaining tissues were excised and frozen^55^.

### Glucose uptake *in vivo*

4 μCi of 2-deoxy-D-[1-^3^H] glucose (2-DG) (PerkinElmer, USA) was mixed in BSA-citrate buffer. A flash injection of radiolabeled mix was administered into the jugular vein of anesthetized (ketamine and xylazine) fed mice at time zero. The specific blood 2-DG clearance was determined by the Somogyi procedure in 25 μl blood samples (tail vein) obtained 1, 15, and 30 min after injection. Tissue samples were removed 30 min after injection. The glucose utilization index was determined by measuring the accumulation of radiolabel compounds. Because values were not corrected by the “discrimination constant” for 2-DG in glucose metabolic pathways, the results are expressed as the index of glucose utilization, in picomoles per milligram of protein per minute^50^,^56^.

### Statistical analysis

All values are expressed as mean ± SEM. Statistical analysis was carried out using the Student-Newman-Keuls test. Statistical analyses were performed using GraphPad Prism version 6.0d (GraphPad Software, USA). Differences were considered significant at ^*^P < 0.05 or ^**^P < 0.01.

## Additional Information

**How to cite this article**: Arce-Cerezo, A. *et al.* HMGA1 overexpression in adipose tissue impairs adipogenesis and prevents diet-induced obesity and insulin resistance. *Sci. Rep.* **5**, 14487; doi: 10.1038/srep14487 (2015).

## Supplementary Material

Supplementary Information

## Figures and Tables

**Table 1:** Serum parameters in wild-type and aP2-HMGA1 transgenic mice fed a HFD.

	HFD	
	Wt	Tg
Triglycerides (mg/dL)	247 ± 31	152 ± 26^**^
FFAs (mmol/L)	0.70 ± 0.06	0.45 ± 0.02^*^
Glycerol (mM)	311 ± 17	207 ± 13^**^
Cholesterol (mmol/L)	5.77 ± 0.3	5.72 ± 0.3
Insulin (ng/ml)	3.62 ± 0.4	3.39 ± 0.4
Glucose (mg/dL)	150 ± 9	155 ± 6
Leptin (ng/mL)	11.6 ± 1.3	5.3 ± 0.9^*^
Resistin (ng/mL)	2.9 ± 0.3	1.3 ± 0.2^*^
Adiponectin (mg/mL)	3.81 ± 0.47	2.07 ± 0.26^*^

Values shown are means ± SEM from at least 6 mice per group. ^*^P ≤ 0.05 and ^**^P ≤ 0.01 *vs.* wild-type mice fed a HFD.
