# Supplementary material for: HMGA1 overexpression in adipose tissue impairs adipogenesis and prevents diet-induced obesity and insulin resistance
Source: Sci Rep. 2015 Sep 28;5:14487. doi: 10.1038/srep14487 (PMC4585969; doi:10.1038/srep14487)

HMGA1 overexpression in adipose tissue impairs adipogenesis and prevents diet-induced obesity and insulin resistance

Altamira Arce-Cerezo[1,2], Miquel García[1,2,3], Aida Rodríguez-Nuevo[1,2], Mireia Crosa-Bonell[1,2], Natalia Enguix[4], Albert Peró[1,2,], Sergio Muñoz[1,2,3], Carles Roca[1,2,3], David Ramos[1,3,5], Sylvie Franckhauser[1,2,3], Ivet Elias[1,2,3], Tura Ferre[1,2,3], Anna Pujol[1,2,3], Jesús Ruberte[1,3,5], Josep A. Villena[3,4], Fàtima Bosch[1,2,3] and Efrén Riu[1,2,3,*].

SUPPLEMENTARY INFORMATION (SI)

Supplementary Figure 1. Whole body effects of HMGA1 overexpression in adipose tissue. A: Adipose tissue weight was measured and plotted as absolute values (*n* = seven mice/group). **B:** Non-adipose tissue weight was measured and normalized by body weight (*n* = seven mice/group). **C:** Non-adipose tissue weight was measured and plotted as absolute values (*n* = seven mice/group). Data represent the mean ± SEM. **P* Ò0.05.

Supplementary Figure 2. Glucose homeostasis in aP2-HMGA1 transgenic mice. A and **B:** Insulin sensitivity was determined after an intraperitoneal injection of insulin (0.75 IU/kg body weight) at the age of 3 and 12 months respectively. Results are calculated as the percentage of initial blood glucose levels (*n* = ten mice/group). **C** and **D:** Glucose tolerance was determined in fasted mice after an intraperitoneal injection of glucose (1 g/kg body weight), and blood glucose levels were measured at the indicated time points at the age of 3 and 12 months respectively (*n* = ten mice/group). Data represent the mean ± SEM. **P* Ò0.05 and ***P* Ò0.01 *vs.* wild-type mice.

Supplementary Figure 3. Gene expression analysis in mature adipocyte and stromal vascular (SVF) fractions of epWAT and BAT. A: *Pref-1* and **B**: *Adiponectin* expression in mature adipocyte and SVF fractions from epWAT of wild-type (Wt) and aP2-HMGA1 transgenic (Tg) mice (*n* = three tissue pools of three mice/pool). **C**: *Pref-1* and **D**: *Adiponectin* expression in mature adipocyte and SVF fractions from epWAT of wild-type (Wt) and aP2-HMGA1 transgenic (Tg) mice (*n* = three tissue pools of three mice/pool). The expression of *Adiponectin* from wild-type mice of adipocytes from epWAT and BAT was used for normalization of *Adiponectin* expression. The expression of *Pref-1* from wild-

type mice of the SVF from epWAT and BAT was used for normalization of *Pref-1* expression. Data are mean ± SEM. **P* Ò0.05 *vs.* wild-type mice.

Supplementary Figure 4. HMGA1 overexpression in iWAT. Gene expression analysis in iWAT. **A:** Downregulated genes in iWAT from transgenic mice fed a HFD (*n* = four mice/group). **B:** Upregulated genes in iWAT from transgenic mice (*n* = four mice/group). **C:** Tissue triglyceride content in iWAT from transgenic mice (*n* = four mice/group). Data are mean ± SEM. **P* Ò0.05 and ***P* Ò0.01 *vs.* wild-type fed a HFD mice.

Supplementary Figure 5. Remodeling process analysis in BAT. A: Representative immunohistochemistry against common ECM components such as lamimin, collagen IV and fibronectin and ECM remodeling proteins such as MMP2 and MMP9 (green) in sections from BAT. Nuclei (blue). Original magnification: 200X. Scale bars: 59.52 µm. **B:** mRNA expression levels of *Cpt1b*, *Lipe* and *Pnpla3* in BAT from wild-type (Wt) and aP2-HMGA1 transgenic (Tg) mice. Data are means ± SEM. **P* Ò0.05 and ***P* Ò0.01 *vs.* wild-type mice.

Supplementary Figure 6. Effects of specific HMGA1 overexpression in adipose tissue on body weight control and gene expression after HFD. A: Body weight (g) from Wild-type and aP2-HMGA1 transgenic mice before and after entering on HFD. 12 week-old mice were placed into the HFD. **B:** Food intake (g/day) results are expressed as daily food intake per mouse body weight (kg) after 10 weeks of HFD (*n* = sixteen mice/group). **C:** Feed efficiency from Wild-type and aP2-HMGA1 transgenic mice after a HFD plotted as: g of body weight/Kcal*100). **D:** Adipose tissue weight was measured and plotted as absolute values (*n* = six mice/group). **E:** Non-adipose tissues weight was measured and normalized by body weight (*n* = six mice/group). **F:** Non-adipose

tissues weight was measured and plotted as absolute values (*n* = six mice/group). **G:** Frequency distribution of adipocyte area in iWAT (*n* = four mice/group). **H:** Gene expression analysis in iWAT from transgenic mice fed a HFD (*n* = four mice/group). **I:** Gene expression analysis in BAT from transgenic mice fed a HFD (*n* = four mice/group). Data are means ± SEM. $^{*}P$ Ò0.05 and $^{**}P$ Ò0.01 *vs.* wild-type mice fed a HFD.

Supplementary Figure 7. Glucose metabolism analysis in transgenic mice after a HFD. A: *In vivo* 2-DG-glucose uptake by iWAT in mice fed a HFD (*n* = five mice/group). **B:** *In vivo* 2-DG-glucose uptake by skeletal muscle (gastrocnemius) in mice fed a HFD (*n* = five mice/group). **C:** Gene expression analysis in skeletal muscle (gastrocnemius) in mice fed a HFD (*n* = four mice/group). Data are means ± SEM. $^{*}P$ Ò0.05 and $^{**}P$ Ò0.01 *vs.* wild-type mice fed a HFD.

Supplementary Figure 8. Western blots from different fat depots blotted with an antibody against HMGA1 and showing a band of approximately 11.5 KDa corresponding to HMGA1. Inset boxes correspond to cropped images observed in Figure 1*D*.

Supplementary Figure 9. Western blots of adipogenic transcription factors and mitochondrial OXPHOS complex proteins in BAT from aP2-HMGA1 transgenic mice. Inset boxes correspond to cropped images observed in Figure 4*D* and *E*.

Supplementary Figure 10. Western blots for phosphorylated Akt (p-AKT) and total Akt (Total AKT) before and after insulin stimulation in epWAT, liver and skeletal muscle. Inset boxes correspond to cropped images observed in Figure 6*C*.

Supplementary Figure 1

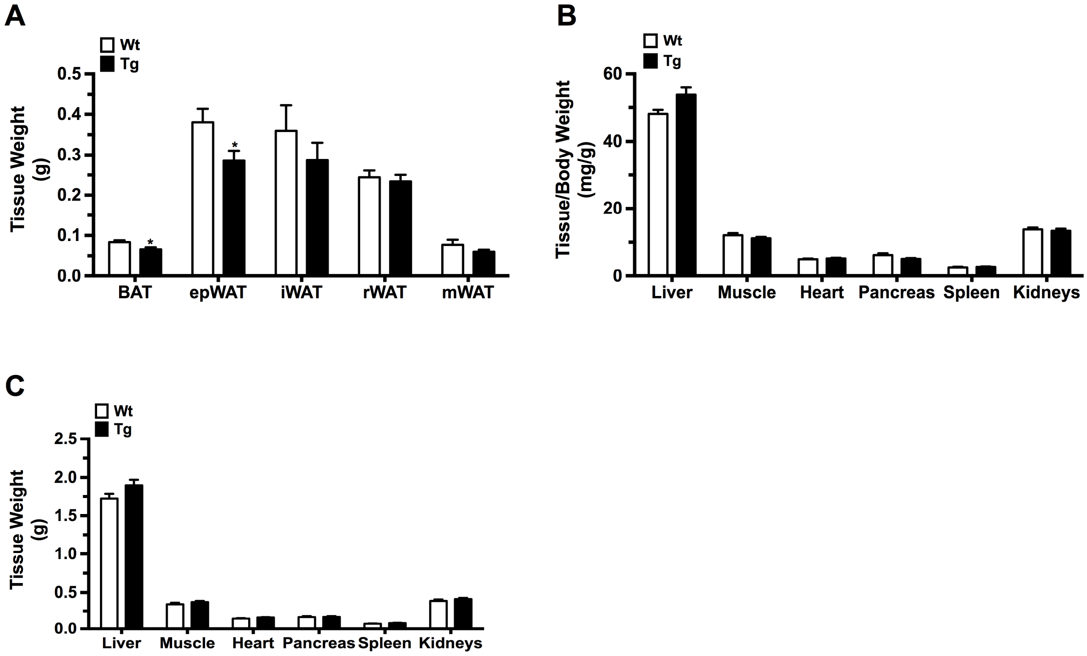

Supplementary Figure 2

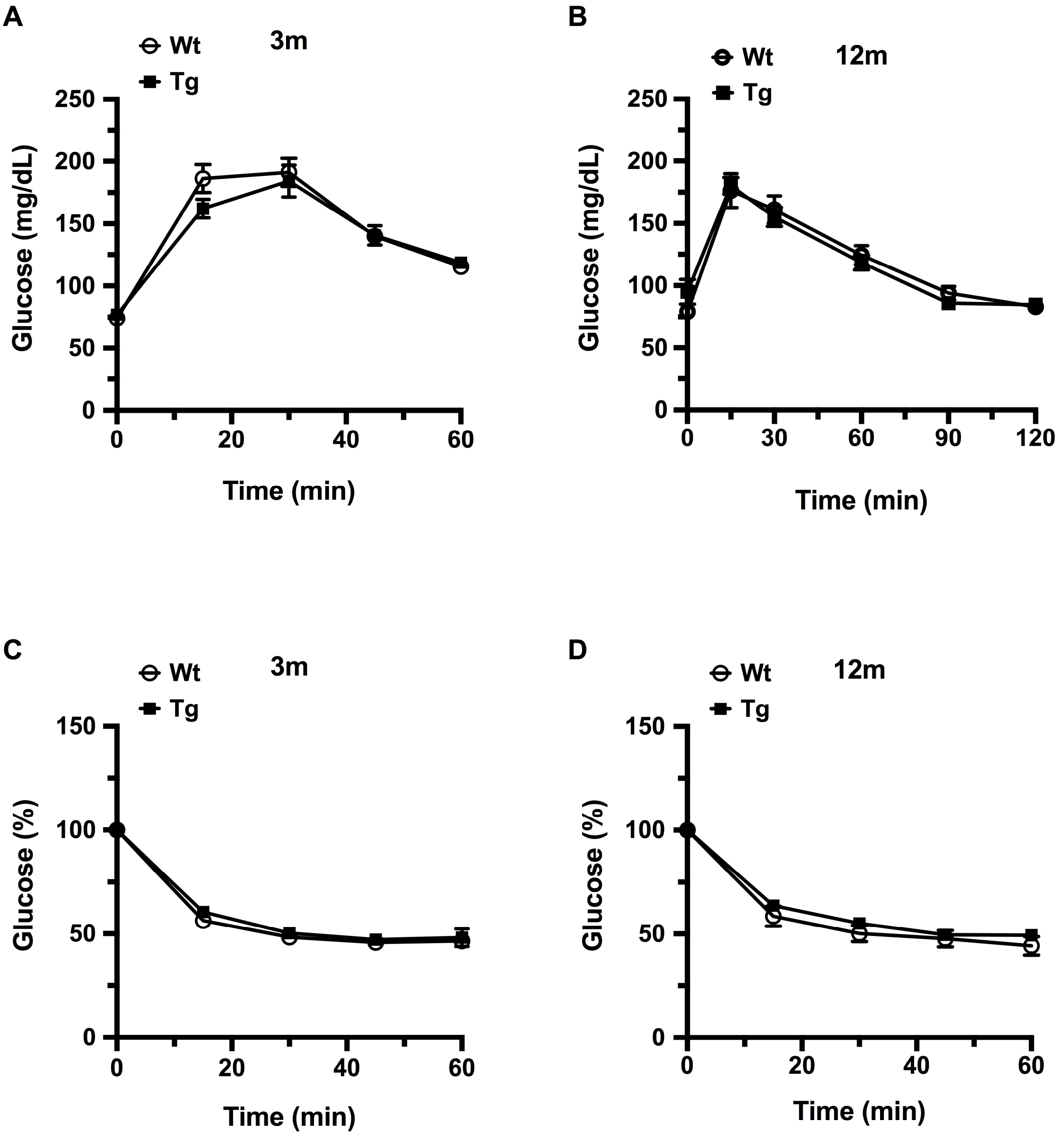

Supplementary Figure 3

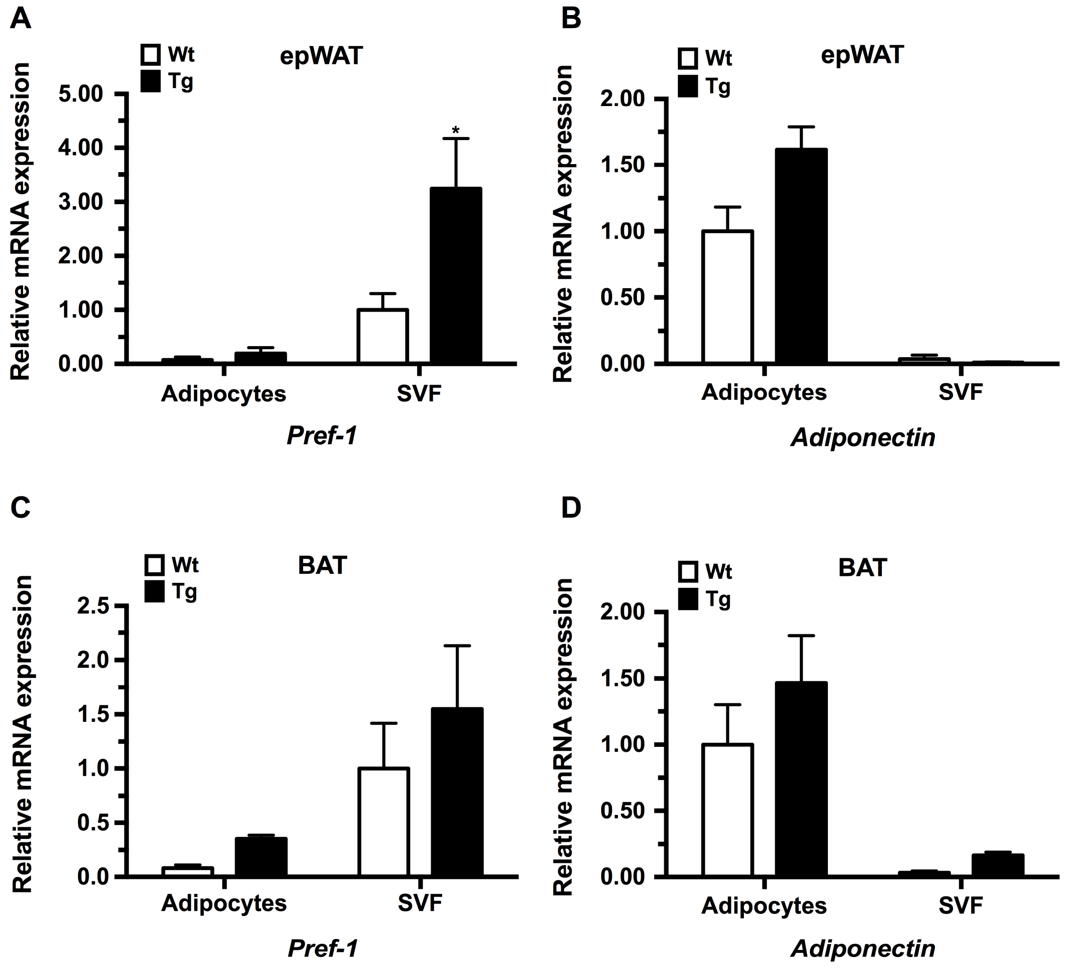

Supplementary Figure 4

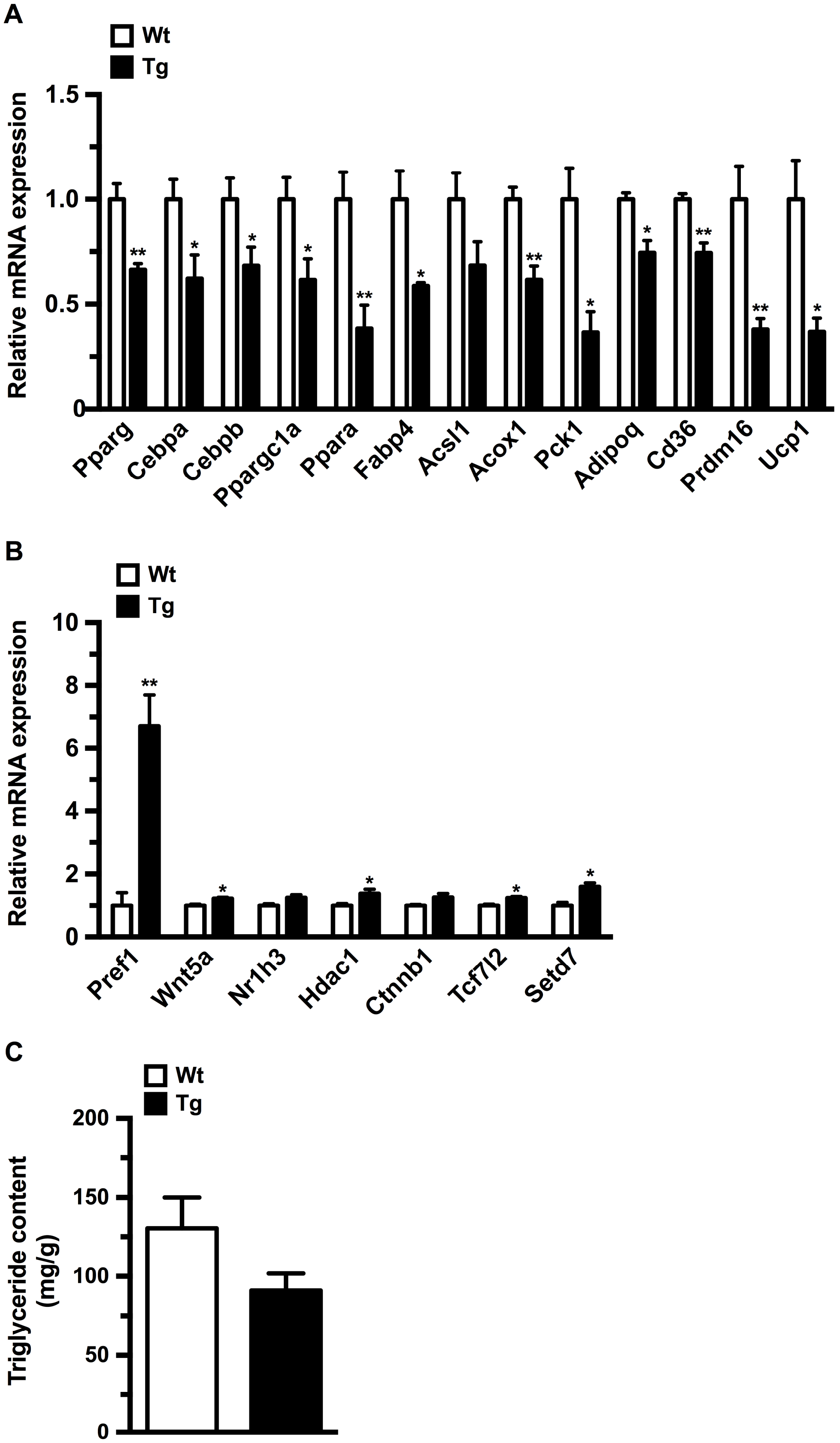

Supplementary Figure 5

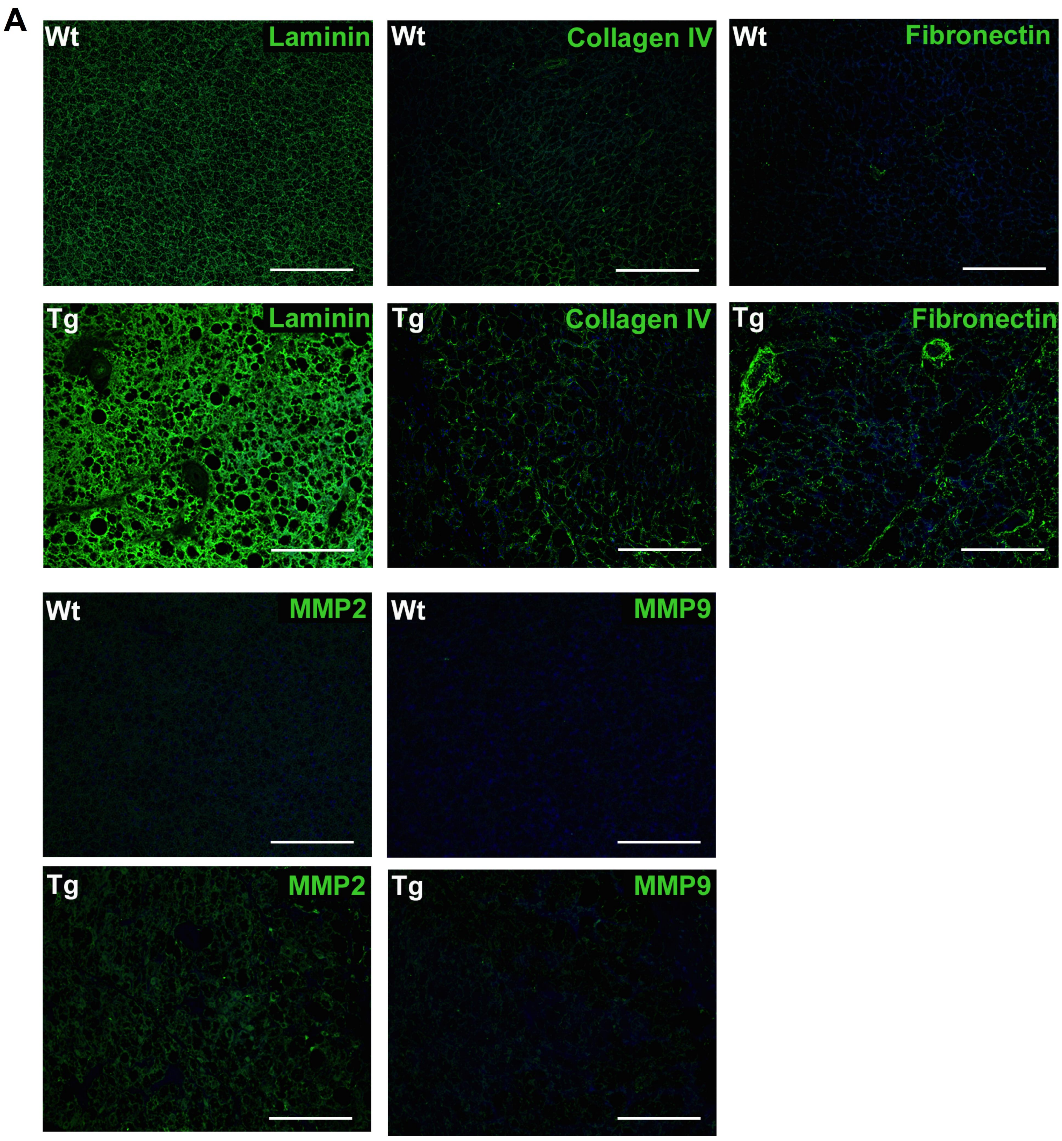

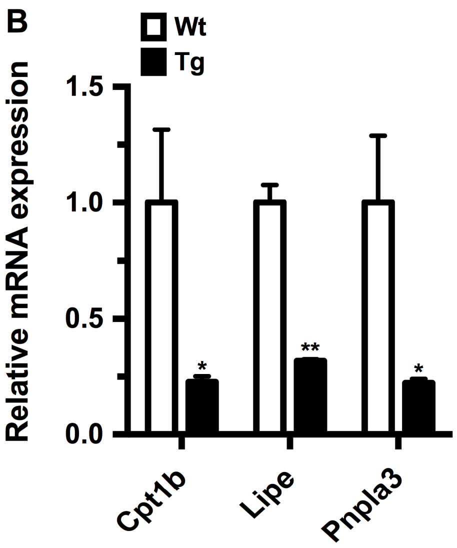

Supplementary Figure 6

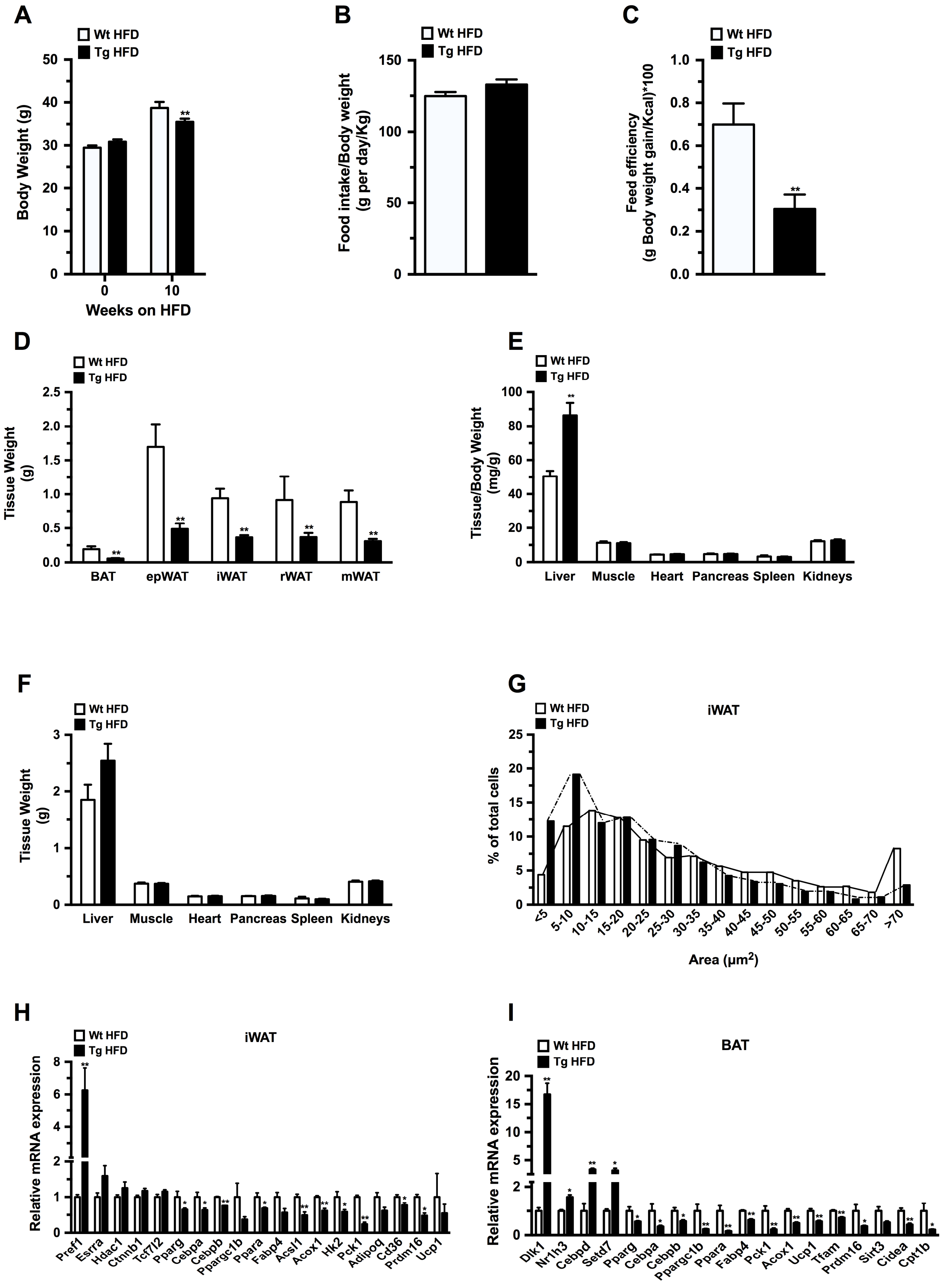

Supplementary Figure 7

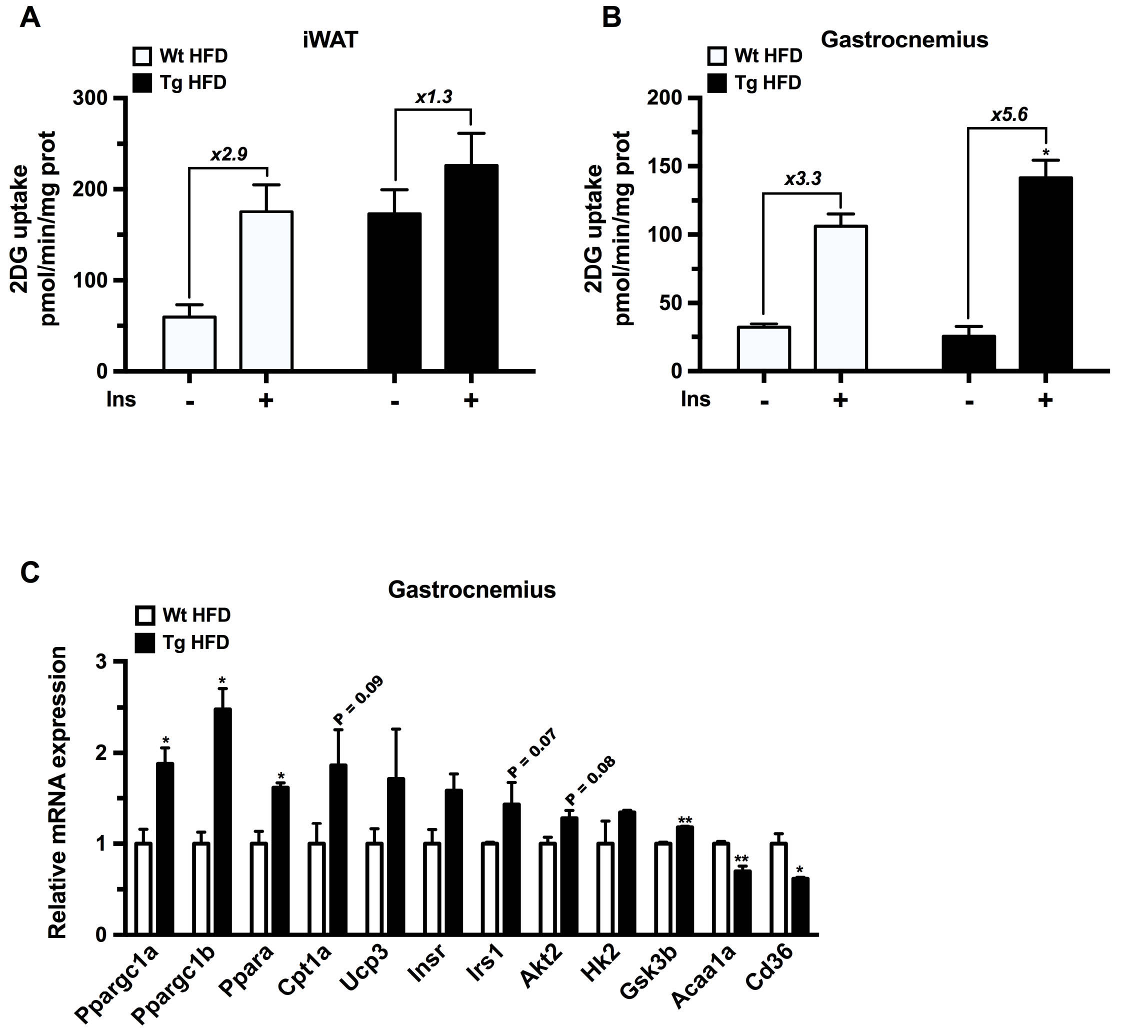

Supplementary Figure 8

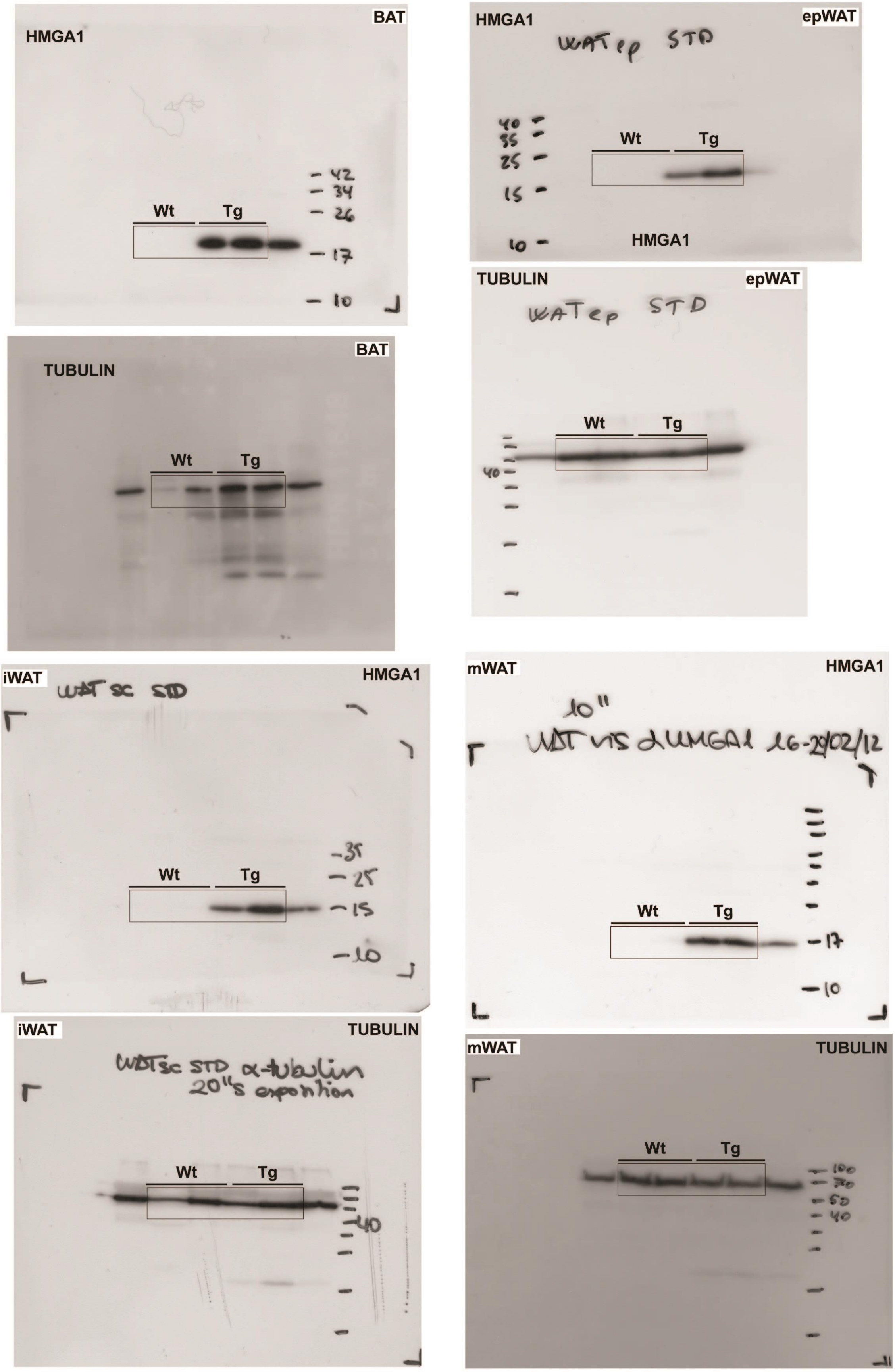

Supplementary Figure 9

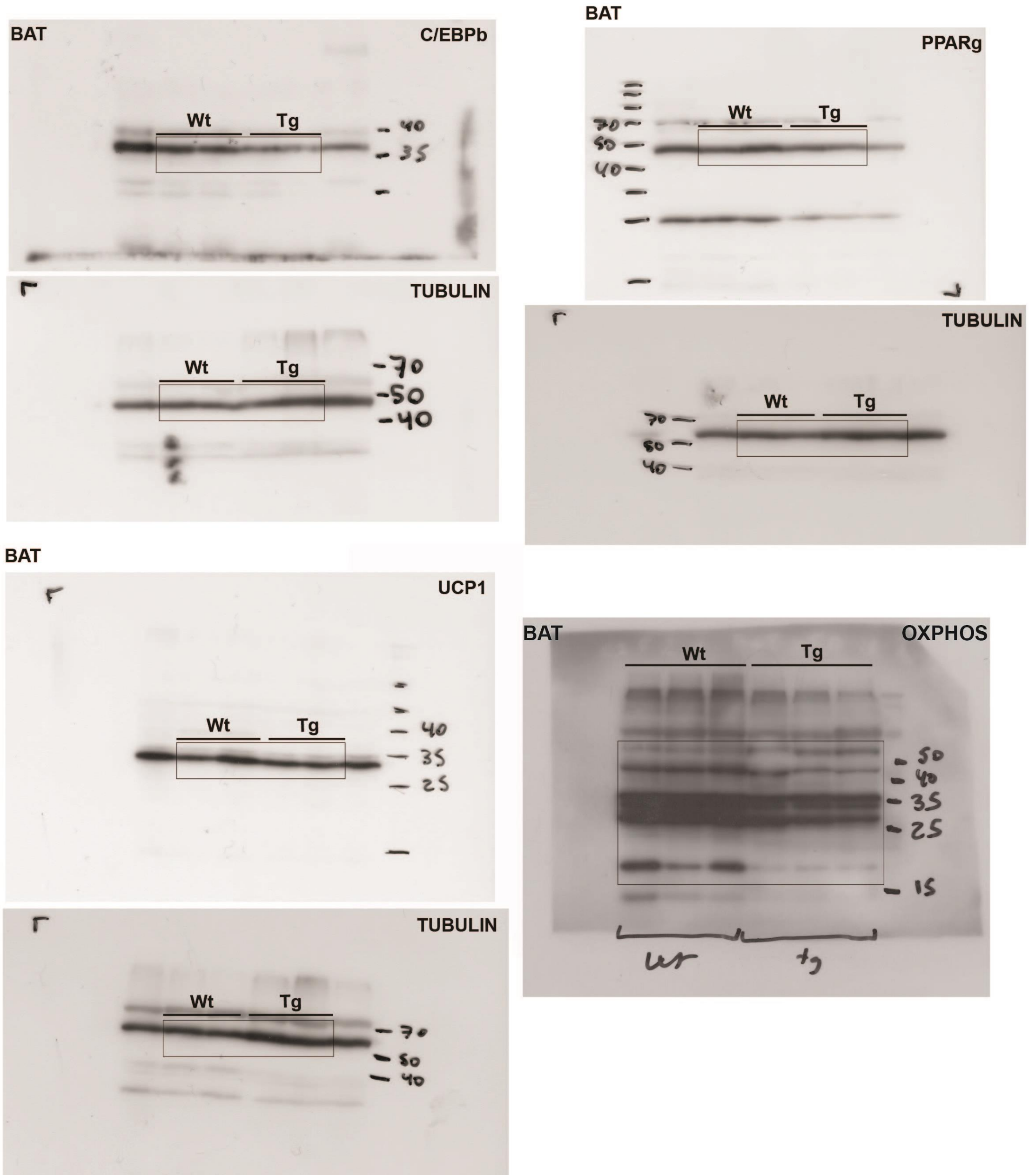

Supplementary Figure 10

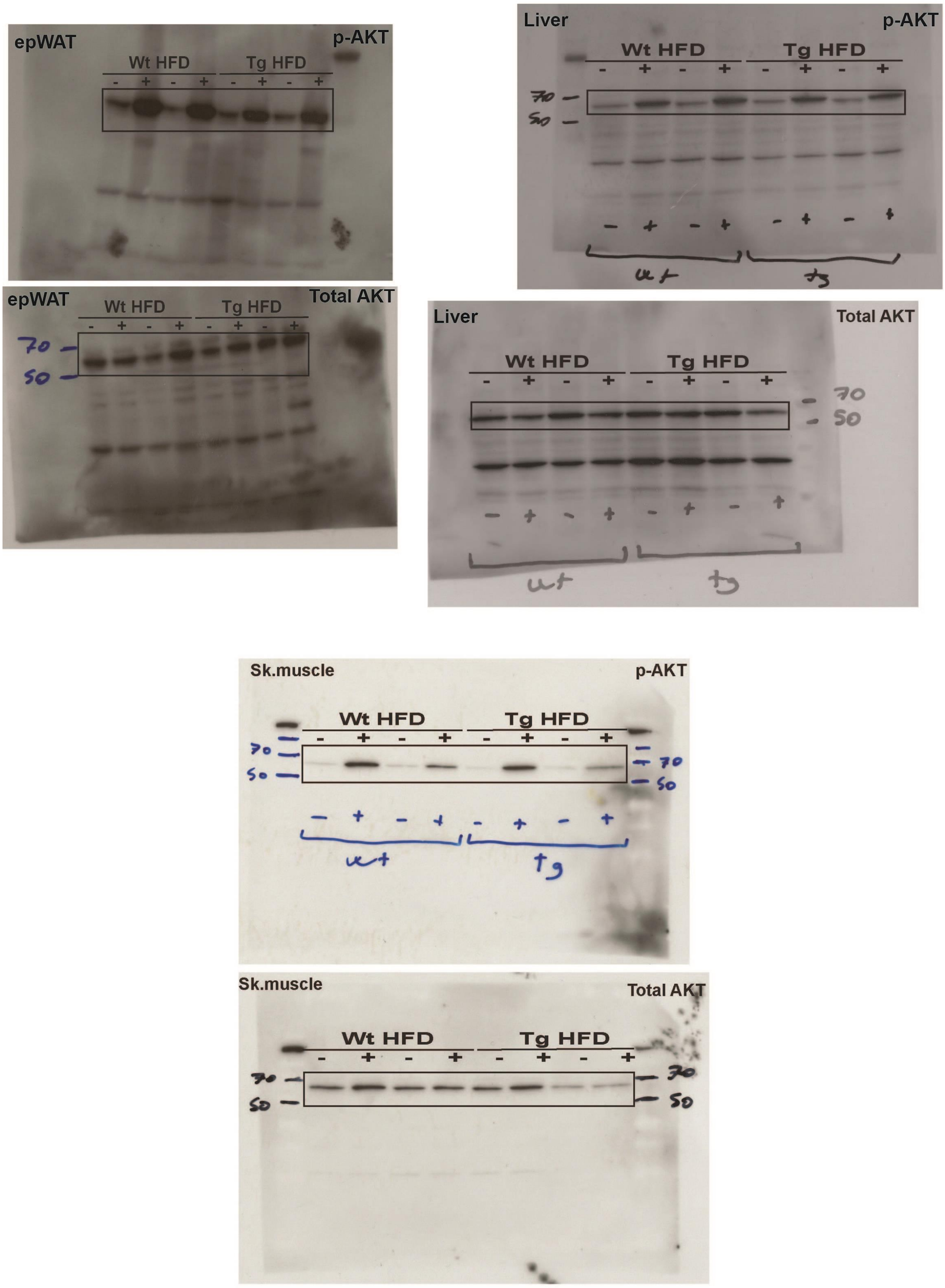

Supplement: Supplementary Information [file srep14487-s1.pdf]
